# Captivity Shapes the Gut Microbiota of Andean Bears: Insights into Health Surveillance

**DOI:** 10.3389/fmicb.2017.01316

**Published:** 2017-07-13

**Authors:** Andrea Borbón-García, Alejandro Reyes, Martha Vives-Flórez, Susana Caballero

**Affiliations:** ^1^Research Group on Computational Biology and Microbial Ecology, Department of Biological Sciences, Universidad de los Andes Bogotá, Colombia; ^2^Max Planck Tandem Group in Computational Biology, Universidad de los Andes Bogotá, Colombia; ^3^Laboratorio de Ecología Molecular de Vertebrados Acuáticos, Department of Biological Sciences, Universidad de los Andes Bogotá, Colombia; ^4^Center for Genome Sciences and Systems Biology, Washington University School of Medicine, Saint Louis MO, United States; ^5^Centro de Investigaciones Microbiológicas, Department of Biological Sciences, Universidad de los Andes Bogotá, Colombia

**Keywords:** 16S rDNA gene, Andean bears conservation, gut microbiota, host–microbiota interactions, herbivory, feeding ecology, metagenomics, *Tremarctos ornatus*

## Abstract

The Andean bear is an endemic species of the tropical Andes who has an almost exclusively plant-based diet. Since herbivorous mammals do not carry enzymes for fiber degradation, the establishment of symbiosis with cellulolytic microorganisms in their gastrointestinal (GI) tract is necessary to help them fulfill their nutritional needs. Furthermore, as described for other mammals, a stable, diverse, and balanced gut microbial composition is an indicator of a healthy status of the host; under disturbances this balance can be lost, leading to potential diseases of the host. The goal of this study was to describe the gut microbiota of wild and captive Andean bears and determine how habitat status influences the composition and diversity of the gut symbiotic community. Fecal samples from wild (*n* = 28) and captive (*n* = 8) Andean bears were collected in “Reserva Pantano de Martos” and “Fundación Bioandina”, Colombia. Composition and diversity analyses were performed using amplicons from the V4 region of the 16S rDNA gene sequenced using the Ion PGM platform. PICRUSt algorithm was applied to predict the gene content of the gut microbiome of wild and captive Andean bears. A total of 5,411 and 838 OTUs were identified for wild and captive bears, respectively. Captive bears contained a lower number of bacterial phyla (*n* = 7) compared to wild individuals (*n* = 9). Proteobacteria (59.03%) and Firmicutes (14.03%) were the phyla that contributed the most to differences between wild and captive bears (overall dissimilarity = 87.72%). At family level, Enterobacteriaceae drove the main differences between the two groups (13.7%). PICRUSt metagenomics predictions suggested a similar pattern of relative abundance of gene families associated with the metabolism of carbohydrates across samples in wild individuals, despite the taxonomic differences of their gut microbiota. Captivity alters the availability and diversity of food resources, which likely reduces microbiota richness and diversity compared to wild individuals. Further considerations should be taken into account for nutritional schemes improving *ex-situ* conservation and its potential as a surveillance tool of endangered populations of wild Andean bears.

## Introduction

The Andean bear has a predominantly plant-based diet, but there is almost no evidence suggesting adaptations for herbivory in this species (Christiansen, 2007, 2008). The gut microbiota greatly influences the energy uptake from the diet in herbivorous, omnivorous, and carnivorous organisms. We hypothesized that the gut microbiota of Andean bears has a composition similar to other herbivorous mammals, with a reduced diversity in captive bears. During the last decades, the habitat of Andean bears has been degraded and the food availability reduced. This altered food availability will likely affect the gut microbiota and the host health. The potential impact of the gut microbiota on the Andean bears’ health indicates the importance of these analyses as a valuable tool into the conservation of the species.

Previous findings with skull morpho-mechanics show no indication of adaptations for herbivory in Andean bears although their mainly herbivorous diet. Evidence shows that bears have a simple digestive tract characterized by a short small intestine, indistinct hindgut, and no cecum, which are features typical of a carnivore’s gastrointestinal (GI) tract (Christiansen, 2007, 2008; Schwab et al., 2011). Since herbivorous mammals do not genetically code for enzymes for fiber degradation, they must establish a symbiosis with cellulolytic microorganisms in their GI tract to help them fulfill their nutritional needs (Flint et al., 2008, 2012; Ley et al., 2008a; Hong et al., 2011; Zhu et al., 2011; Shepherd et al., 2012; Ilmberger et al., 2014; Xue et al., 2015). This symbiotic process comprises a complex gut microbial consortium that converts indigestible polysaccharides from plants into short chain fatty acids (SCFAs) that are absorbed by the intestine into the bloodstream to provide energy to the host (Bergman, 1990; Dill-Mcfarland et al., 2015). Previous research has shown that diversity, structure, and function of gut microbiota change mainly in response to diet adaptation (Ley et al., 2008a,b; Zhu et al., 2011). For this reason, the gut microbiota associated and GI physiology of herbivorous mammals is well specialized, dominated by several cellulolytic and obligate anaerobic lineages of bacteria, allowing energy uptake and nutrient absorption from a highly fibrous diet to the host (Zhu et al., 2011; Ilmberger et al., 2014). The gut microbiota of herbivorous mammals is also differentiated from omnivore and carnivore mammals, which is dominated by facultative anaerobes (Ley et al., 2008a; Zhu et al., 2011).

The predominantly herbivorous habit of Andean bears is believed to be a consequence of a diet shift from carnivory towards herbivory after the Great American Biotic Interchange, allowing avoidance of competition with other large carnivores. Therefore, this is a relatively recent evolutionary process which has not allowed the species to develop a specialized physiology and morphology for exploiting these kinds of resources (Soibelzon and Schubert, 2011). Within the bear group there are other examples of dietary specialization to herbivory without physiological adaptations. One such example is the Giant Panda, where studies have demonstrated the existence of several groups of bacteria that allow them to consume bamboo (Zhu et al., 2011; Li et al., 2014; Xue et al., 2015). Other evidence of microbiome specialization in the Giant Panda revealed the presence of genes associated with the *Clostridium* group, with cellulolytic and other carbohydrate hydrolytic activity (Zhu et al., 2011; Tun et al., 2014). Since Andean bears are closely related to Panda bears, and also have different plant-associated nutritional challenges, we hypothesized that the Andean bear would also form symbiosis with specific microbes adapted for the metabolism of complex carbohydrates.

In addition to microbial adaptations for host specific dietary restrictions, host–microbiota interactions are crucial for host physiology and health status, including immunity, metabolism and behavior (Zhu et al., 2011; Subramanian et al., 2014; Hird et al., 2015; Dill-McFarland et al., 2016). There is increasing evidence suggesting that alterations of the gut microbiota (dysbiosis) can affect host health, leading to disease development. In humans, these alterations have been associated with diabetes, inflammatory bowel disease (IBD), obesity, rheumatoid arthritis, and susceptibility to infections, among others (Kinross et al., 2011; Nicholson et al., 2012; Cheng et al., 2015). Furthermore, due to the lack of specialized mechanisms and structures for herbivory (i.e., chambered stomach, specialized small intestine, and modified cecum and colon), the microbial composition of the GI tract greatly influences the health and nutritional status of the host; hence, it is very sensitive to dietary changes and stressors under disturbed habitats which can lead to dysbiosis and disease development (Amato et al., 2013; Barelli et al., 2015; Stumpf et al., 2016). Reduction of diversity in gut microbiota commonly shows evidence of dysbiosis and it may imply the loss or decrease of microbial functional groups, making the microbiome less efficient, less resilient and more susceptible to pathogens (Cho and Blaser, 2012; Nicholson et al., 2012; Amato et al., 2013; Waite and Taylor, 2015).

The Andean bear is an endemic species of the tropical Andes and is cataloged as Vulnerable by the International Union for the Conservation of Nature (IUCN) (Goldstein et al., 2008). Human-bear conflict and habitat degradation are two main causes of the decrease of Andean bear populations. Principally, this decrease occurs as retaliatory killing due to cattle predation by bears as a consequence of population isolation (Jorgenson and Sandoval-A, 1997; Goldstein et al., 1999; Goldstein, 2002; Kattan et al., 2004). However, the Andean bear, which depends on more than 114 plant species, consume cattle only rarely and opportunistically, not as an active strategy of foraging, but because of the expansion of human villages into the Andean bear habitat (Jorgenson and Sandoval-A, 1997; Goldstein et al., 1999; Kattan et al., 2004).

This habitat degradation that Andean bears are facing could have a strong effect on gut microbial diversity because habitat degradation leads to reduction of plant diversity and the availability of food plants (Amato et al., 2013). These small populations with a high conservation value are particularly vulnerable to habitat degradation, pathogens and dietary stressors (Schwab et al., 2011). In the Northern Andes, priority conservation actions for Andean bears have been implemented by local and international organizations and NGOs. Another important threat to Andean bear conservation is illegal capture and trading. When bears are confiscated, there are important efforts to rehabilitate and reintroduce them into their natural habitat. However, dietary management of these animals in captivity may affect their microbiome capacity for nutrient uptake potentially leading to an unbalanced microbial community (especially for cubs). The nutritional management of bears in captivity has been empirically made based only on a daily caloric goal, supplemented primarily with domesticated fruits and cereals, usually oatmeal (Schwab et al., 2011). This diet is not based on a wild bear diet, and the reduced diversity of the diet likely causes a functional reduction of gut microbes and loss of microbial diversity (Amato et al., 2013; Barelli et al., 2015). Reduced microbial diversity can make the host less resistant to disturbances and more susceptible to potentially pathogenic microorganisms (Schwab et al., 2009, 2011; Amato et al., 2013; Barelli et al., 2015).

In this study, we aimed to characterize the gut microbial diversity of wild and captive Andean bears, and understand how the dietary management during captivity influence changes in the microbiome composition. We hypothesized that, similar to other herbivorous bears, Andean bears have a modified gut microbiota with functional groups related to the breakdown of complex carbohydrates. We also expected a diversity reduction in the gut microbiota of captive Andean bears. This assessment provides the first findings describing the gut microbiota of Andean bears and its relationship to their diet.

## Materials and Methods

### Collection of Fecal Samples

**FIGURE 1:**
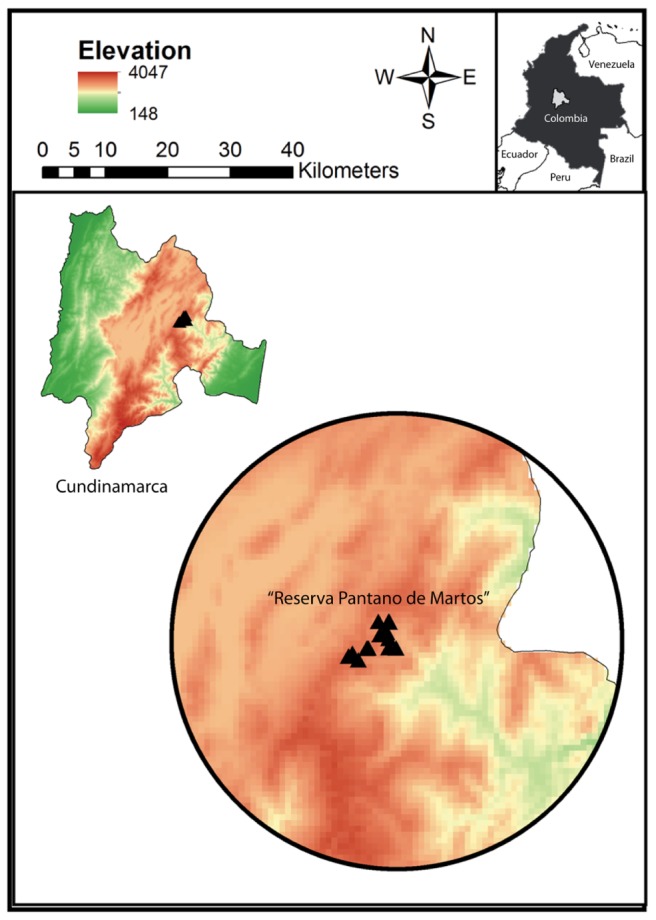
Study site “Reserva Pantano de Martos” is a protected area with low levels of disturbance by human economic activities. It is located in Guatavita, Cundinamarca (Colombia) and encompasses páramo and high Andean forest ecosystems. Each triangle corresponds to individual sample locations and all the samples were collected at a mean elevation of 3000 m.a.s.l.

Samples from wild and captive Andean bears were collected during a period of 2 years between 2013 and 2015. Twenty-eight fecal samples from wild Andean bears were collected in “Reserva Pantano de Martos”, Guatavita, Colombia (**Figure 1**) together with eight fecal samples from captive Andean bears housed in “Fundación Bioandina,” including four males and four females. The eight captive individuals came from several confiscation processes in separate locations in Colombia and after that, they were kept in very similar conditions with no variable diet regimes. The population of wild bears was monitored using camera traps. Data such as pictures and videos of the time the bears were moving through different paths, were used to guarantee that the samples came from different individuals. The samples were obtained from the inner part of the feces, in an effort to avoid cross-contamination with the environment. For captive animals, samples were collected and transported to the lab within 24 h of deposition, while for wild individuals; samples were collected only when evidence supporting less than 4 days of deposition was obtained. Samples were transported at 4°C and stored in the laboratory at -80°C until further processing. For every sample from captive bears, health status (disease incidence, antibiotic use, stereotypic behavior, etc.) was registered. Sampling of captive animals was performed with the corresponding permit from “Fundación Bioandina” administration and samples were collected by the internal veterinarian. Wild samples were donated by the environmental authority CAR (Corporación Autónoma Regional de Cundinamarca) and collected in company of representative personal of the CAR.

### DNA Extraction and 16S rDNA Amplification

Genomic DNA was extracted using the QIAamp DNA Stool Mini Kit (QIAGEN) according to the manufacturer recommendations. DNA concentration was measured with Qubit^®^ and stored at -20°C until use. Afterward, the V4 hypervariable region of the 16S rDNA was amplified using primers 515F: 5′ GTGCCAGCMGCCGCGGTAA and 806R: 5′ GGACTACHVGGGTWTCTAAT (Caporaso et al., 2010a). The PCR reaction contained: 1X Buffer, 0.6 μg/μL of BSA, 0.2 mM of dNTPs, 1.5 mM of MgCl2, 0.3 mM of each primer, 2 U/μL of Taq polymerase and 3 μL of template DNA. The reaction profile was set up as follows: Initial denaturation at 94°C for 5 min; 19 cycles of 94°C for 45 s, 55°C for 45 s and 72°C for 45 s; Final extension at 72°C for 7 min. The PCR products were visualized with Gelred^®^ in a 1.5% (w/v) agarose gel and purified with QIAquick PCR Purification Kit (QIAGEN). Quantification of amplified DNA was carried out using Qubit (Invitrogen) and stored at -20°C until use.

### Library Preparation and Sequencing

Amplification of the V4 region was possible for sixteen fecal samples from wild bears (due to low DNA quality of twelve samples from wild bears) and eight fecal samples from captive bears. Those 24 samples were used to prepare amplicon libraries, using P1 Ion PGM compatible adapters and different barcodes for each sample (Ion Xpress Barcodes Kit, Life Technologies). Library quantification was performed with a 7500 Real-Time PCR System (Applied Biosystems), template preparation and emulsion PCR were carried out with Ion One Touch (Life Technologies). Finally, only thirteen samples from wild bears and eight samples from captive bears yielded sufficient DNA templates for further sequencing, which were loaded on a 318X and a 316X chip, respectively, and sequencing was performed on the Ion PGM platform (Life Technologies). The DNA sequences were placed in the ENA under the study accession number PRJEB21054.

### Bioinformatic and Statistical Analyses

FastQC analysis (Andrews, 2010) was performed to assess the quality of the obtained raw reads and to establish the quality threshold for further filtering steps. Following, an adapters removal and quality filtering of reads was performed using Trimmomatic (Bolger et al., 2014) with the following parameters: quality trimming with a sliding window of 4 pb and quality threshold of 20, and final length length cutoff 100 pb. Samples with less than 300 reads per individual were removed, reducing the dataset to a final number of five wild and eight captive animals. QIIME v1.9.1 (Caporaso et al., 2010b) was used for OTU clustering at 97% similarity using the UCLUST algorithm, taxonomic assignment against the Greengenes Database version 13_5, estimates of α diversity (Phylogenetic Diversity, Observed OTUs, Chao1, Shannon, Simpson) and β diversity (Bray–Curtis, weighted and unweighted UniFrac). For β diversity, OTUs with a relative abundance lower than 1% and present in less than two individuals were removed. Bray–Curtis, unweighted and weighted UniFrac distances were used to compare between status (captive and wild) and between captive females and males’ gut microbiota. The OTUs with no assignable taxonomy in the Greengenes Database, were further taxonomically annotated using BLAST algorithm against the nucleotide database of NCBI. Sequences with no match with *e*-values below 1*e*-10 and identity above 90%, and no clear alignment to other 16S rDNA sequences, were removed as they likely reflect either high sequencing error rate or low level of genomic DNA contamination. Afterward, assigned OTUs were filtered out for sequences present in only one sample and below the 0.01% relative abundance. The resulting taxonomy, of the sequences not assigned by QIIME was visualized using MEGAN6 (Huson et al., 2016) to compare with the taxonomic pattern obtained for the assigned OTUs with the Greengenes Database.

In order to have insights into the metabolic potential of the gut microbiota of wild and captive Andean bears, we used PICRUSt v.1.0.1 (Phylogenetic Investigation of Communities by Reconstruction of Unobserved States) (Langille et al., 2013) with the 16S rDNA dataset under default settings. PICRUSt predicts the functional profile of a microbial community based on the 16S rDNA profile, using an extended ancestral-state algorithm that predicts the gene content from a marker gene survey, using an existing database of reference microbial genomes, generating an annotated table of predicted KEGG orthologs (KOs) counts for each sample. The abundance of each OTU was normalized using the copy number of the 16S rDNA operons identified in its corresponding genome found by PICRUSt. Afterward, the complete metagenome was predicted using the Greengenes database version 13_5. To assess the accuracy of the PICRUSt predictions, the Nearest Sequenced Taxon Index (NSTI) was calculated for each sample. Using PRIMER-E (Clarke and Gorley, 2006), an ANOSIM test was used to determine if there were significant differences between groups using a square-root transformed Bray–Curtis dissimilary matrix between groups, and SIMPER analysis was used to determine the taxa that account for major similarities between captive and wild status. A Welch’s *t*-test was implemented using STAMP (Parks et al., 2014) to compare the relative abundance of the predicted KEGG pathways and OTUs for captive and wild statuses.

## Results

We characterized the gut microbiota of captive Andean bears maintained in Guasca and Mesitas, and wild Andean bears located in Pantano de Martos, Guatavita, Colombia. The sampled wild population has been estimated to harbor up to 18 individuals, which feed on an enormous variety of food plants. In contrast, captive Andean bears are feed on a limited variety of food resources, which do not correspond to their diet in the wild.

**FIGURE 2:**
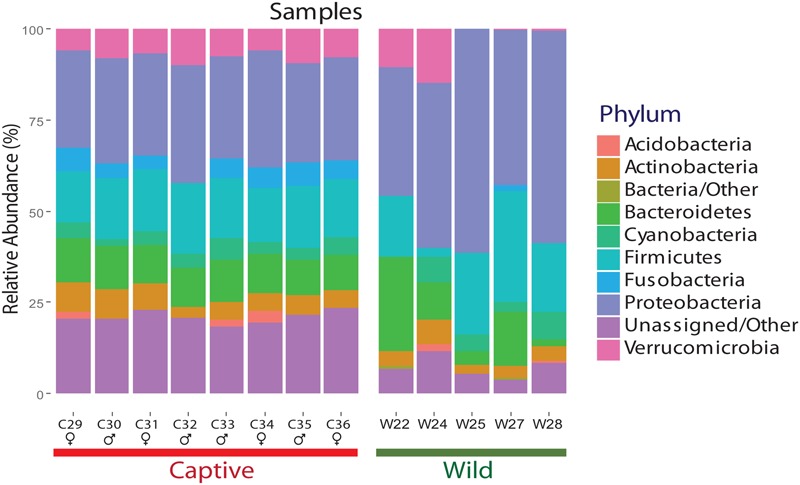
Relative abundance of each phylum for wild and captive Andean bears gut microbiota. Per phylum relative abundance for samples from wild and captive Andean bears. Proteobacteria, Bacteroidetes, and Firmicutes are the most abundant phyla for both statuses. Moreover, an average of 2.05 and 24.4% of reads for wild and captive samples, respectively, were not assigned to any taxa within the domain Bacteria. It is an evident pattern of evenness for captive bears’ samples whereas more variation in the composition of gut microbiota for wild bears’ samples is observed. Proteobacteria is the most abundant phylum for both statuses.

The final dataset contained five wild and eight captive samples. The five wild samples belonged to the rainy tropical season in the same year. Clustering analysis generated a total of 5411 OTUs for wild individuals and 838 OTUs for captive individuals, using a similarity threshold of 97%. Significant differences were found between captive and wild individuals (*R* = 0.88, *p*-value = 0.001, ANOSIM test). For both captive and wild individuals, Proteobacteria, Bacteroidetes, and Firmicutes were the main phyla shaping the gut microbial diversity. However, a reduction in the number of phyla in the gut microbial community was observed in captive individuals (*n* = 7 phyla) compared with wild individuals (*n* = 9 phyla) (**Figure 2**). The diversity of wild bears’ gut microbiota was higher than in captive bears according to mean Shannon (wild: 4.87 ± 1.11 vs. captivity: 3.23 ± 0.15; *p*-value 0.00196, ANOVA test), Chao1 (wild: 944.66 ± 1.1 vs. captivity: 19.02 ± 1.11; *p*-value 0.0618, ANOVA test) and Simpson (wild: 0.90 ± 0.10 vs. captivity: 0.86 ± 0.10; *p*-value 0.354, ANOVA test) estimates of α diversity.

**FIGURE 3:**
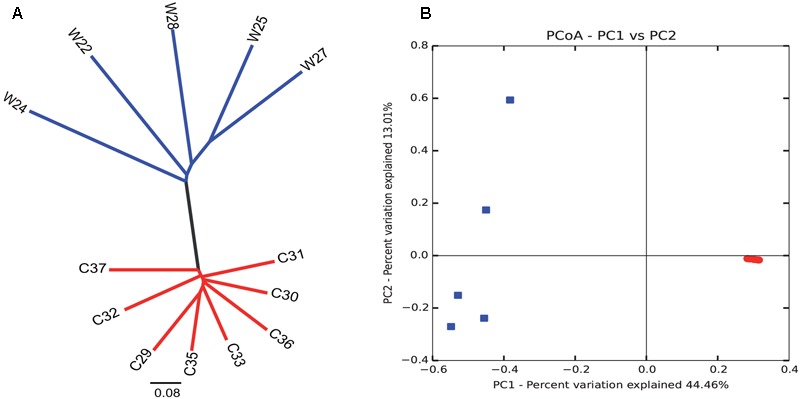
Intra- and inter-group variations highlight the reduction in diversity among captive individuals compared to wild animals. Clustering analysis **(A)** and PCoA graphical representation **(B)** based on Bray–Curtis similarity showed a clear grouping pattern that separates wild individuals from captive individuals. It is remarkable that intra-group variations are highly reduced for the captive group. Contrastingly, wild individuals group evidence a more heterogeneous gut microbiota composition. Each dot in **(B)** corresponds to each sample of captive (red) and wild (blue) sample.

Bray–Curtis dissimilarity analysis between wild and captive individuals showed that the gut microbiota of wild Andean bears has an uneven composition across samples, with higher intra-group variations (average similarity = 74.89%), contrasting with an even composition for captive Andean bear samples, with lower intra-group variation (average similarity = 96%) (**Figure 3**). There was no identifiable core of shared OTUs between all captive and wild bears, only one OTU was shared in 75% of the samples, with no assigned taxonomy using Greengenes and NCBI nt databases. Moreover, among the wild bears, only two OTUs appeared in 80% or more of the samples, consisting of *Sphingobacterium faecium* and *Pseudomonas* sp. In contrast, there was a core microbiota consisting of nine OTUs for 100% of captive individuals, none of which were assigned to any available sequence in the Greengenes database, but only one of them was assigned to *Pseudomonas helmanticensis* using BLAST against the nucleotide Database of NCBI (100% identity and 98.82% coverage).

**FIGURE 4:**
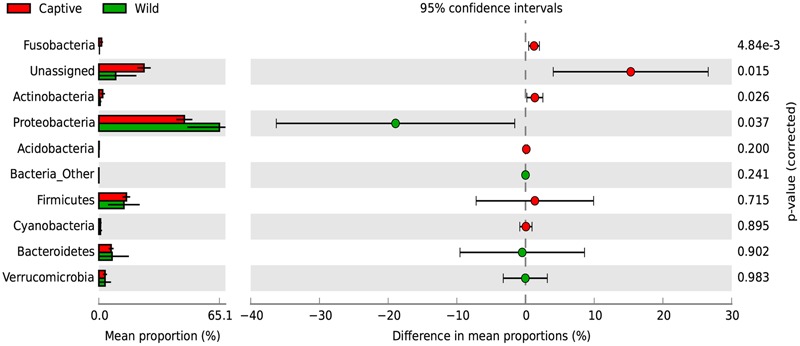
Changes in relative abundance of identified phyla determining the diversity of wild and captive Andean bears. Extended error bar plot identifying significant differences between mean proportions of bacterial phyla in captive (red) and wild (green) Andean bears, Corrected *p*-values are shown at the right. It is noticeable that a high proportion of reads from captive samples belonged to the Unassigned group, and were significantly higher than unassigned reads for wild Andean bears (*p* < 0.001, Welch’s *t*-test). In addition, Actinobacteria and Fusobacteria were slightly higher in captive individuals (*p* < 0.05, Welch’s *t*-test). Proteobacteria was significantly higher in wild individuals compared with captive individuals (*p* = 0.017, Welch’s *t*-test).

A SIMPER analysis was conducted to determine which taxa explained the dissimilarities between wild and captive status, contributing to the observed patterns of taxonomic composition and possibly to the functional variations that could be found between captive and wild individuals. This analysis calculates for each taxon, its percentage contribution to the (dis)similarities between groups. At the phylum level, changes in the relative abundance of Proteobacteria (33.26%), Firmicutes (12.03%) and Bacteroidetes (7.71%) are the main contributors for diversity differences observed, where there was an overall dissimilarity of 92.71% between captive and wild individuals. Within all the identified phyla for captive and wild individuals, Fusobacteria and Actinobacteria were more prevalent in captive individuals (*p* < 0.01 and *p* = 0.026, respectively, Welch’s *t*-test), and Proteobacteria was more abundant in wild individuals (*p* = 0.017, Welch’s *t*-test) (**Figure 4** and Supplementary Table 1). Also, a high proportion of the Unassigned Bacteria contributes greatly to the differences between captive and wild individuals, although these OTUs do not necessarily belong to the same phyla, making any inference about their phylogenetic diversity and ecological role within the gut microbiota difficult. Families and genera contributing to the main differences between captive and wild individuals are listed in **Table 1**.

**Table 1:** Families and genera accounting for major differences between wild and captive bears gut microbiota.

Phylum	Genus	Contribution (%)	Mean wild	Mean captivity
Proteobacteria	Enterobacteriaceae/Other	13,7	9730	11,2
Unassigned	Unassigned	12,06	883	97,1
Proteobacteria	Comamonadaceae/Other	7,688	6000	9,2
Proteobacteria	*Pseudomonas*	5,421	2540	2,7
Firmicutes	*Paenibacillus*	4,452	1110	20,5
Bacteroidetes	*Sphingobacterium*	2,147	385	13,4
Firmicutes	Clostridiaceae/Other	1,754	452	6,3
Bacteroidetes	*Pedobacter*	1,729	457	2,2
Proteobacteria	Caulobacteraceae/Other	1,551	283	5,8
Firmicutes	*Coprococcus*	1,54	271	2,4

*Mean abundances are shown for both wild and captivity groups of individuals.*

The subset of Unassigned OTUs (*n* = 1852) was retrieved and analyzed separately showing clear differentiation between captive and wild bears (Supplementary Figure 1). Afterward, those OTUs were annotated by using BLAST against the nucleotide database of NCBI. Only 26.18% of the reads were taxonomically assigned within the Bacteria domain. However, after filtering out the low abundance OTUs and singletons, a total of 169 OTUs remained, among the 132 from the captive dataseet, one had a taxonomic assignment to Ochrobactrum rhizosphaerae (89.47% identity and 99.4% coverage) and from the 37 from the wild samples, only six OTUs from wild samples were assigned, five of those belonged to Proteobacteria (91.25–100% identity and 98.9–99.2% coverage) and one to Bacteroidetes (89.93% identity and 99.3% coverage).

ANOSIM test suggested that gender of captive individuals is not a determining factor shaping differences in gut microbiota (*R* = 0.38, *p* = 0.002, ANOSIM test). Furthermore, a SIMPER analysis showed that males and females gut microbiota have only 8.09% of dissimilarity, and the changes of abundance of Verrucomicrobia (13.52%), Fusobacteria (13.40%), and Proteobacteria (40.23%) were the main contributors to the differences among genders.

Ruminococcaceae and Lachnospiraceae are important families of the phylum Firmicutes, associated with a healthy GI tract in mammals, but are also abundant in herbivorous species, due to their capacity of degradation of complex sugars (Barelli et al., 2015). We identified such families in wild samples, but they were below detection levels in samples from captive bears, which could be related with a lower prevalence, below the sensitivity levels of the sequencing strategy implemented in the study. Other families of the phylum Firmicutes and their changes in relative abundance are listed in **Table 2**.

**Table 2:** Changes in the relative abundance of glycosyl-hydrolase containing families within Firmicutes phylum identified as present in either Wild or Captive animals.

	Wild	Captivity
Paenibacillaceae	0.25	6 × 10^-3^
Clostridiaceae	0.09	3 × 10^-3^
Ruminococcaceae	8.6 × 10^-4^	0
Lactobacillaceae	0.22	9.9 × 10^-4^
Lachnospiraceae	0.38	0

**FIGURE 5:**
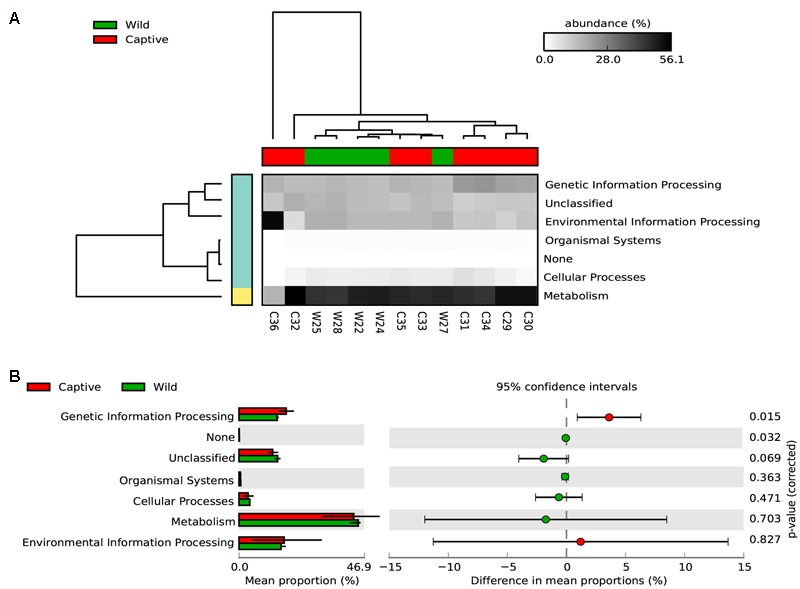
Predicted KEGG pathways using PICRUSt for wild and captive Andean bears. **(A)** The heatmap shows that most of the predicted gene families belong to metabolism pathways, followed by genetic information processing and environmental information processing. The dendrograms at the upper and left to the heatmap were constructed using the UPGMA algorithm and based on Bray–Curtis distances between samples. **(B)** Extended error bar plot showing the differences in mean proportions between wild and captive individuals for each KEGG Metabolism Pathway and its associated corrected *p*-value at the right (Welch’s *t*-test).

To assess how the metabolic potential of Andean bears changes under different environments, we applied the PICRUSt algorithm. To assess the accuracy of these predictions, the NSTI was calculated. This index quantifies the availability of nearby genome representatives for each microbiome sample, and indicates the average divergence between each OTU and the reference genome in the Greengenes database (Langille et al., 2013). We obtained an average NSTI of 0.06 ± 0.01 and 0.17 ± 0.12 for wild and captive samples, respectively, which are within the previously estimated ranges for non-human mammals (Langille et al., 2013) and are coherent with scores obtained for similar studies (Mao et al., 2015; Sullam et al., 2015). These low values suggest accurate predictions for molecular functions of microbial communities in the GI tract of wild and captive Andean bears. The analysis showed a similar pattern of relative abundance of gene families associated with the metabolism of carbohydrates across samples in wild individuals, despite the taxonomic differences of their gut microbiota (Supplementary Figure 2). Furthermore, there was a similar gene content for both statuses. It was predicted that 46.94 and 46.65% of the gene families for wild and captive individuals, respectively, belonged to KEGG metabolism pathways. The only exception was sample C36, a sample corresponding to a 6 months captive cub (other samples were adults), which has different assignment of predicted genes, with a higher gene content related to environmental information processing pathways and lower for metabolism pathways, with only one gene family associated with carbohydrates metabolism (**Figure 5**).

**FIGURE 6:**
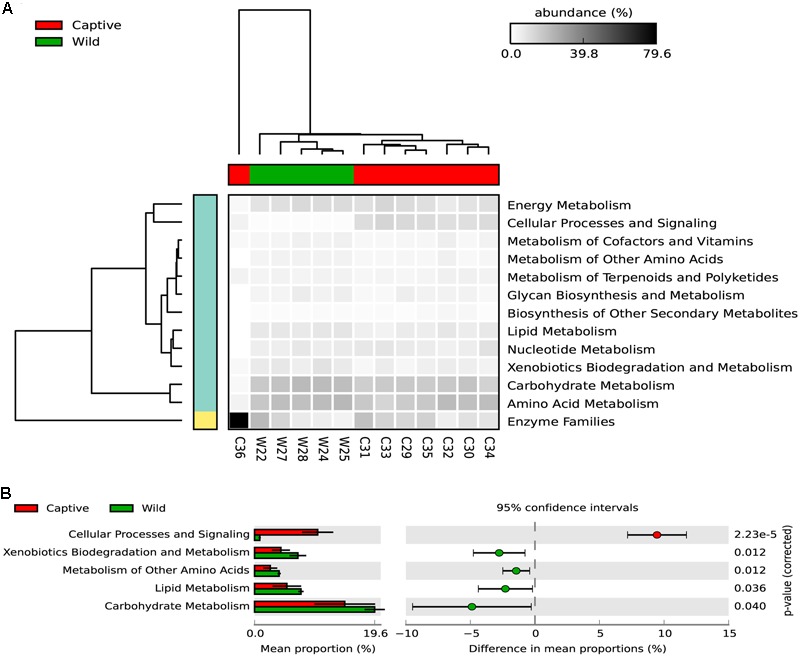
**(A)** The heatmap shows the relative abundance of each KEGG metabolism pathway across the wild and captive samples. The dendogram in the upper part of the heatmap shows the grouping pattern of samples based on Bray-Curtis distances between samples, this separates captive from wild status, excepting the C36 samples, which belongs to a six-years old cub. **(B)** An extended error bar plot indicating the differences of mean proportion for each predicted metabolic pathway occurring in the gut microbiome of captive and wild Andean bears. Proportions means are the average predicted amount of genes based on OTUs abundance and normalization by predicted 16S rDNA number of operons. The gene count is high for both statuses but tended to be higher for captive individuals (*p* < 0.05, Welch’s *t*-test).

Regarding the metabolic pathways, 157 gene families were predicted among captive and wild individuals (**Figure 6** and Supplementary Table 2). Moreover, there were significant differences in the predicted abundance of carbohydrate metabolism (Bonferroni corrected *p*-value: 0.039, Welch’s *t*-test), cellular processes and signaling (Bonferroni corrected *p*-value: 2.22*e*-5, Welch’s *t*-test), lipid metabolism (Bonferroni corrected *p*-value: 0.0360, Welch’s *t*-test), metabolism of other amino acids (Bonferroni corrected *p*-value: 0.012, Welch’s *t*-test), and xenobiotics biodegradation and metabolism pathways (corrected Bonferroni *p*-value: 0.012, Welch’s *t*-test). For these pathways, the mean relative abundance was higher in wild individuals.

Xenobiotics biodegradation and metabolism was significantly different between captive and wild individuals, being slightly lower for captive individuals. In total, 20 gene families were predicted for both groups, and a clustering pattern was observed separating wild from captive individuals. Within these gene families, four were significantly higher in wild Andean bears compared with captive Andean bears (Supplementary Figure 3 and Table 3).

## Discussion

The gut microbiota of Andean bears varied significantly between wild and captive individuals. Bacterial diversity was higher in wild individuals and lower for individuals living in captivity. These findings are congruent with previous evidence, which has demonstrated that perturbed habitat as well as captivity conditions act as stressors and are correlated with decreased gut microbial diversity (Amato et al., 2013; Barelli et al., 2015; Bennett et al., 2016). The difference in similarity patterns observed for wild and captive individuals could be explained by changes in diet. Captive individuals have all the same assigned diet, one plausible explanation why their gut microbiota displayed a very similar composition among all the individuals.

Wild individuals can use the habitat differentially, moving through long distances and across a wide elevation gradient according to flowering, fruit development seasonality, and availability patterns of plants (Amanzo et al., 2007; García-Rangel, 2008; Peyton, 2008; Figueroa, 2013). This foraging behavior may explain the observed dissimilarity between samples, given that the host’s living conditions are not constant throughout the year in all the areas where Andean bears are feeding. Interestingly, it was only found one shared OTU in 75% of the individuals within the wild Andean bear group, whereas several OTUs were identified in all the captive Andean bear samples.

We observed that regardless of the geographic origin of captive individuals, all of them exhibited a very similar gut microbiota, with slight variations in relative abundances of microbial lineages. In contrast, wild individuals from the same geographic area, showed marked differences in gut microbiota. These data suggest a strong influence of habitat use, where diet is the most likely driver of the observed changes. Captive individuals receive almost the same diet, but wild individuals can feed on different resources depending on availability and movements across their home ranges (Peyton, 2008; García-Rangel, 2012; Figueroa, 2013). Previous evidence from Asiatic black bears, has shown that biogeography of captive individuals (feed on a standard zoo diet) appears to be a main factor influencing differences between individuals (Song et al., 2017). In this study, captive Andean bears were maintained in enclosures within the same ecosystem, sharing the same location and climatic conditions, and limited variation was displayed between individuals. Only two bears (cubs) were in enclosures with different geographic and climatic features, and still no significant differences were found. Further analyses should address the differences for individuals with different geographic origins maintained under contrasting geoclimatic conditions.

Lachnospiraceae and Ruminococcaceae are two of the most common bacterial families within the Firmicutes phylum found in the gut environment of several organisms (Dill-Mcfarland et al., 2015). It has been hypothesized that they play a role in maintaining digestive health, since they are active producers of butyrate, crucial for the health of colonic epithelial tissue. Moreover, they have an important role as active plant degraders. Their importance relies on the harboring of key-carbohydrate enzymes, sugar transport mechanisms and other metabolic features that make them specialist groups for degradation of recalcitrant and complex plant material (Bergman, 1990; Dill-Mcfarland et al., 2015; Xue et al., 2015). Interestingly, they were only found in wild Andean bear samples and below detection levels for samples from captive individuals, which could suggest that the fiber-reduced diet in captivity is modifying the ability of the gut microbiota to degrade recalcitrant substrates such as cellulose, hemicellulose, lignocellulose, among others, that are commonly found on the main resources of wild Andean bears’ diet. This functional adjustment has been observed for other species under several scenarios of habitat quality (Amato et al., 2013; Barelli et al., 2015) and constitutes an interesting consideration about how the degradation of habitat and its consequent reduction of food resources might trigger the decline of important metabolic pathways associated with nutrients use. This represents a valuable argument for revising and implementing monitoring plans of wild and captive individuals’ health, particularly when cubs are rehabilitated with the idea to reintroduce them to the wild.

These findings could be important for cubs, which are expected to have lower diversity in the gut microbiota compared with juveniles and adults (Spor et al., 2011; McKenney et al., 2015). Among the threats for Andean bears conservation, illegal trafficking of cubs and separation from their mother by local villagers, have in fact a tremendous impact on the gut microbiota at maturity, because breastfeeding provides a route of vertical transmission of microbes and the secondary step of the intestinal epithelium colonization after birth (Funkhouser and Bordenstein, 2013; Mueller et al., 2015). Further sampling through time could provide information about the variability of gut microbiota as well as the establishment of this community during cub development. This could be important for individuals raised in captivity, where the vertical transmission of microbes via breastfeeding is reduced or absent in some cases, which could have negative outcomes on the gut microbiota assemblage.

As well as Lachnospiraceae and Ruminococcaceae, Firmicutes and Actinobacteria have been widely associated with cellulases important for the plant cell wall degradation (Berlemont and Martiny, 2013). However, previous research has shown that Proteobacteria, Bacteroidetes, and Verrucomicrobia (among other phyla) contain species that have genes encoding putative glycosyl-hydrolases for cellulose utilization and can potentially metabolize it (Berlemont and Martiny, 2013). In addition, it has been revealed that several bacterial genera that were previously unrecognized by their ability to metabolize complex carbohydrates such as cellulose, hemicellulose, lignocellulose, etc., have in fact genes encoding for glycosyl-hydrolases that allow them to degrade these recalcitrant substrates (Medie et al., 2012; Soares et al., 2012).

We predicted several gene families associated with carbohydrate metabolism. More than 50% of such gene families were related to carbohydrate metabolism, lipid metabolism, xenobiotics metabolism, energy metabolism, etc., suggesting the importance of these gut microbiota’s functions for the digestive proficiency of the host. This core of metabolic capacities shared between the captive and wild individuals has been observed for other wild species belonging to unperturbed and perturbed habitats (Barelli et al., 2015). However, as in other reports, we found significant differences for some KEGG categories, which could be explained by the different environmental conditions that Andean bears face in captivity and the wild. Interestingly, it was found that Xenobiotics biodegradation and metabolism gene families’ abundances generated two differentiated clusters for wild and captive individuals, which could be linked with the environmental conditions in which these groups live. Since wild Andean bears have a plant rich diet, it is expected to find molecules related with defense mechanisms against herbivores, which could include terpenoids, tanines, and other compounds that can be toxic for the Andean bear. These core functions in the Andean bear gut microbiota could be involved with detoxifying xenobiotics present in the plant-based diet of the host, as it has been reported for the gut microbiota of other mammals (Barelli et al., 2015). In contrast, in captivity the diet is composed of fruits, cereals and meat, which cannot act as a selective pressure for detoxification-related genes in the gut microbiome.

A high proportion of reads were not assigned to any bacterial phylum, which could suggest tremendous undescribed and unknown diversity in the gut microbiota of Andean bears, but also could be associated with short reads, which prevented us from identifying a significant match with taxa in public databases. For this reason, we repeated the taxonomic assignment using BLAST against the nucleotide database of the NCBI, and we still found a substantial proportion of unassigned reads to any domain, only a small percentage was assigned to Bacterial taxa, and the composition pattern did not differ from the one obtained based on Greengenes. This evidence suggest that the gut microbiota of Andean bears is still an unknown environment. Some studies describing the gut microbiota of other bears species (i.e., black bears, grizzly bears, polar bears, and panda bears), regardless of their diet, have reported that Firmicutes and Proteobacteria are the most common phyla (Glad et al., 2010; Schwab et al., 2011; Zhu et al., 2011; Xue et al., 2015; Song et al., 2017), being consistent with our results. However, our data suggest a higher prevalence of Proteobacteria than Firmicutes and comparison between studies should be made carefully, since they used different sequencing strategies that could be an important confounding factor to make conclusive comparisons with our data.

Despite 16S rDNA analysis giving an approximation to the taxonomical composition of a microbial community, it does not give information about their metabolic potential, thus, limiting our conclusions. However, 16S rDNA analysis constitutes a valuable and cost-efficient approach for surveillance and monitoring wild populations as well as captive individuals. It not only provides researchers with insights regarding the abundance of potential beneficial bacteria, but also allows screening and detecting the occurrence of pathogens and their potential transmission in captivity. In contrast, PICRUSt predictions are a suitable proxy to understand the functioning of a microbial community, but its accuracy relies on the availability of closely related annotated bacterial genomes in databases, and the phylogenetic distances between the organisms in the microbial community and the reference genomes used by the PICRUSt algorithm. Although the calculated NSTI values suggest a suitable prediction, this does not represent a 100% of correlation between predicted genes and the real metagenome of this community, and there is still uncertainty about the genetic potential of this microbiome. The Andean bear gut microbiota is still an unexplored and unknown environment, which could harbor taxa with previously unknown metabolic capabilities. For this reason, further metagenomic approaches would allow us to understand the metabolic importance of the Andean bears’ gut microbiota for this feeding ecology.

The gut microbiota is a dynamic community. Time and field conditions can drive changes in the gut microbiota and deviate related-conclusions (Hale et al., 2016). Conditions such as the exposure time after defecation of fecal samples in the wild can be a potential source of variation in the observed composition and diversity. According to Hale et al. (2016), the gut microbiota diversity and the OTU composition change after 12 h of deposition of wild fecal samples. For wild Andean bears, it is challenging to ensure an exposure time below 12 h, since is not easy to follow individual animals. However, we used some criteria such as temperature, moisture, and consistency of the sample to guarantee that fecal samples were not exposed for more than 4 days. We also took the inner part of the sample to ensure an almost anaerobic environment and to avoid cross-contamination with transient microorganisms. Considerations about intra-group (i.e., captivity, wild) variation related to taxonomic profiles and diversity patterns should be taken into account for further comparisons. For instance, several strict anaerobes of the GI tract can be underestimated in our analysis because of these may get lost during exposure.

The study of the gut microbiota of wild species is a promising field on conservation research, because it provides an important source of information related with the health status of individuals, and may serve for the monitoring and surveillance of entire populations (Stumpf et al., 2016). Also, it can reflect the effect of diets designed for captive individuals, where the low diversity of resources can act as a stressor, leading the individual to dysbiosis and further disease development. Microbiome biology is still a poorly explored field in conservation biology and has an enormous potential for elucidating the effects of habitat degradation, lower resource availability, population isolation, and captivity maintenance conditions on host health. Microbiome analyses could be a powerful tool for government policy makers, improving the current management plans of emblematic and threatened wild species such as the Andean bear, whose populations have been reduced and little is known about the current health status of populations.

## Author Contributions

AB-G, SC, AR, and MV-F planned the study and the experimental design. AB-G performed the sample collection and preservation, DNA extraction, amplification, library preparation and sequencing. AB-G and AR performed analyses and interpreted results. AB-G, AR, SC, and MV-F wrote and edited the manuscript. All authors gave final approval for publication.

## Conflict of Interest Statement

The authors declare that the research was conducted in the absence of any commercial or financial relationships that could be construed as a potential conflict of interest.
