# Supplementary material for: Captivity Shapes the Gut Microbiota of Andean Bears: Insights into Health Surveillance
Source: Front Microbiol. 2017 Jul 13;8:1316. doi: 10.3389/fmicb.2017.01316 (PMC5507997; doi:10.3389/fmicb.2017.01316)
Supplement: Supplementary file 1 [file Presentation_1.PDF]

Supplementary Material

Captivity maintenance shapes the gut microbiota of Andean bears: insights into health surveillance

Andrea Borbón-García*, Alejandro Reyes, Martha Vives-Flórez, Susana Caballero

*** Correspondence:** Andrea Borbón: ad.borbon174@uniandes.edu.co

Supplementary Fig. 1. Heatmap showing the differences between captive and wild samples based on the Unassigned OTUs using the Greengenes Database. The subset of Unassigned OTUs was retrieved and analyzed separately. OTUs with a relative abundance below 0.1% were filtered out. An UPGMA clustering was performed on the Bray-Curtis distances based on the relative abundance profiles among samples.

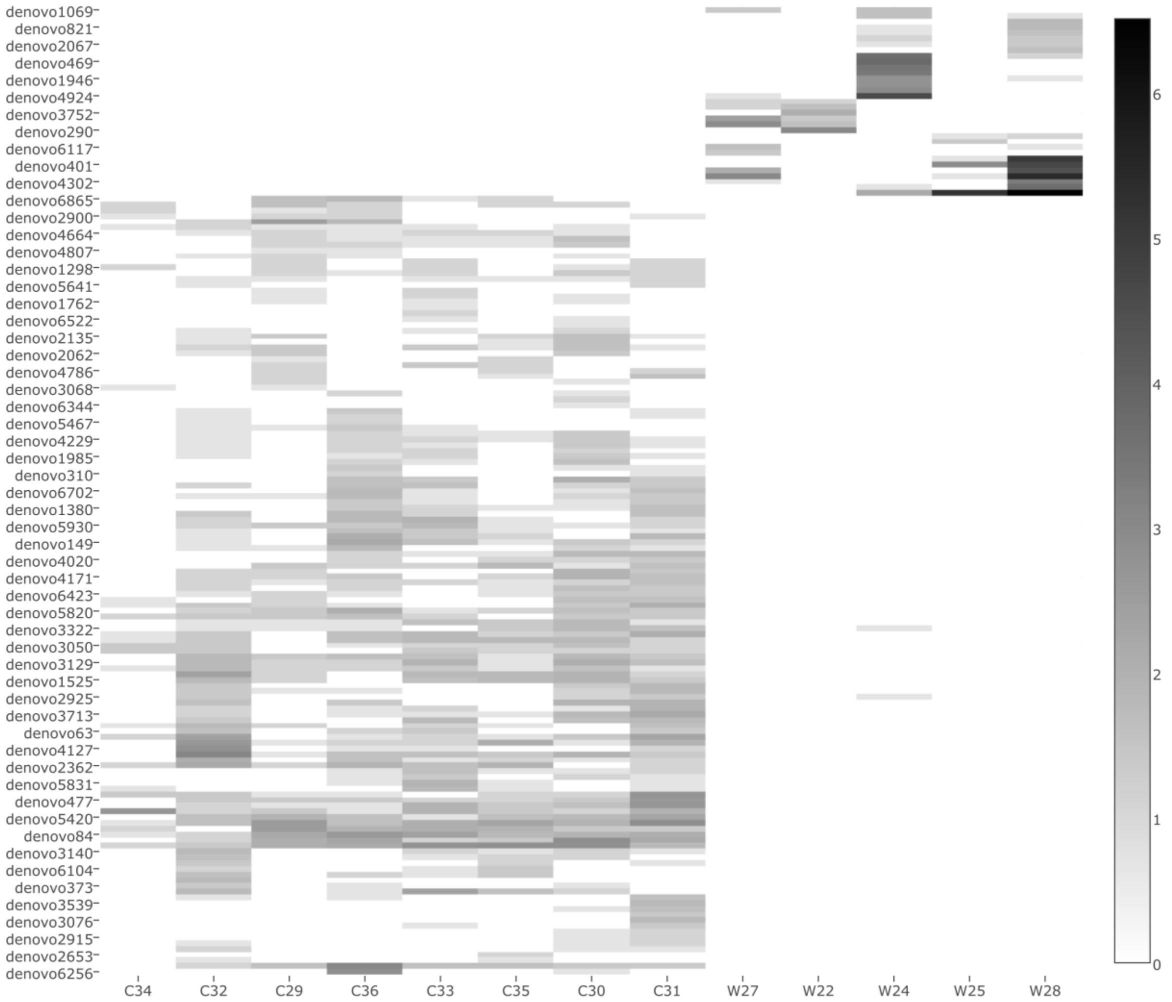

Supplementary Fig. 2. Stacked plot showing the variations of predicted gene families for carbohydrate metabolism between wild and captive samples. Regardless the taxonomic patterns and heterogeneity between wild samples compared with captive samples, it is evident that metabolic functions are kept constant for all the samples, which means that despite the taxonomic differences, specific conditions of this environment are shaping this microbial community metabolism in a similar way. Sample C36 came from a cub (6 months), and only twenty gene families were predicted, within these, only one geme family was found, corresponding to glycolysis and gluconeogenesis pathways.

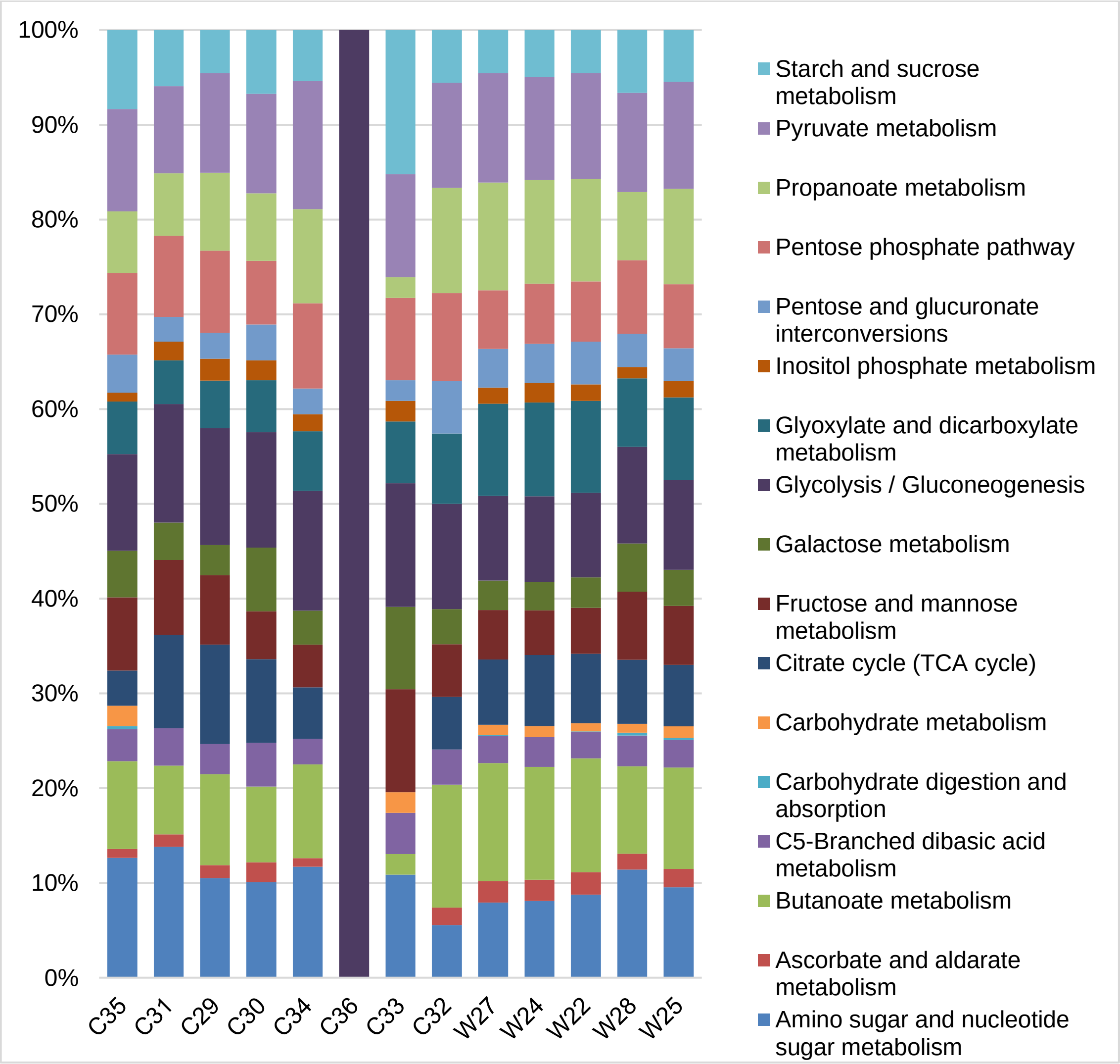

Supplementary Fig. 3. Heatmap showing the changes in relative abundance of predicted genes associated with xenobiotic biodegradation and metabolism pathways. A total of 20 gene families were predicted to be present in the gut microbial community of captive and wild Andean bears. An UPGMA clustering was performed on the Bray-Curtis distances based on the relative abundance profiles among samples and pathways, dendrograms are shown at left and above the heatmap.

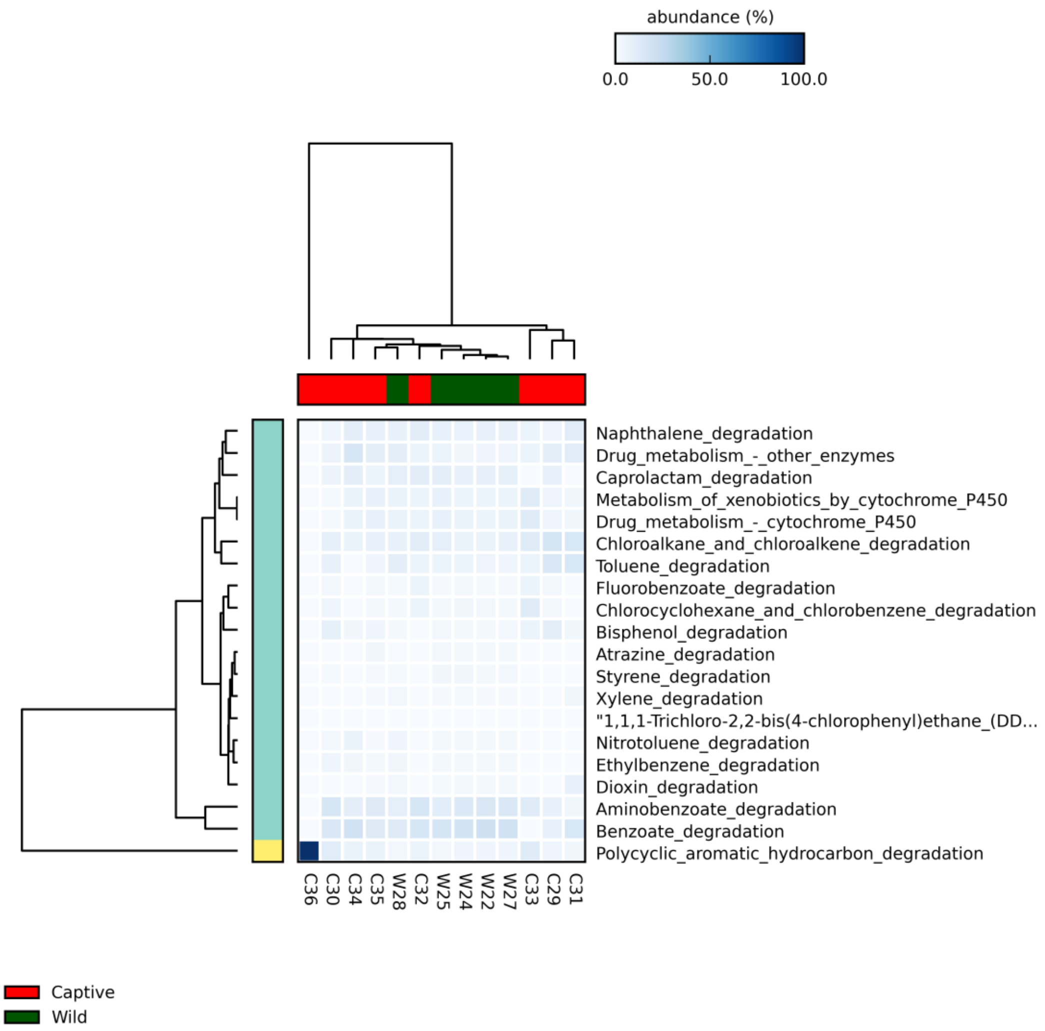

Supplementary Table 1. Welch's t-test for identified phyla in captive and wild Andean bear samples. *P*-values were corrected for multiple hypothesis testing using Bonferroni correction. *P*-value < 0.05: *, <0.01: **. Differences in mean relative abundance of each phylum and its standard deviation are shown.

Phylum	Captive		Wild			
	Mean rel. freq. (%)	±S.D.	Mean rel. freq. (%)	±S.D.	Difference between means	p-values (corrected)
k__Bacteria;Other	3.14	2.87	0.14	0.22	3.00	0.04*
k__Bacteria;p__Acidobacteria	0	0	0.22	0.33	-0.22	0.24
k__Bacteria;p__Actinobacteria	0	0	4.23	2.07	-4.24	0.026*
k__Bacteria;p__Bacteroidetes	0	0	11.30	9.19	-11.30	0.81
k__Bacteria;p__Cyanobacteria	0	0	4.91	3.37	-4.91	0.76
k__Bacteria;p__Firmicutes	0	0	18.56	9.35	-18.56	0.69
k__Bacteria;p__Proteobacteria	1.37	2.18	51.77	9.14	-50.40	0.017*
k__Bacteria;p__Fusobacteria	1.44	0.79	0.02	0.04	1.43	2.04e-3**
k__Bacteria;p__Verrucomicrobia	0	0	4.47	5.35	-4.47	0.17
Unassigned;Other	24.40	3.59	2.05	1.70	22.34	3.39E-8**

Supplementary Table 2. Welch's t-test for predicted KEGG pathways associated with metabolism in captive and wild Andean bear samples. *P*-values were corrected for multiple hypothesis testing using Bonferroni correction. *P*-value < 0.05: *, <0.01: **. Differences in mean relative abundance of each metabolic pathway and its standard deviation are shown.

KEGG Pathways	Captive		Wild			
	**Mean relative frequency (%)**	**±S.D.**	**Mean relative frequency (%)**	**±S.D.**	**Difference between means**	**p-values (corrected)**
Amino Acid Metabolism	16.14	0.83	20.12	0.30	-3.98	0.088
Biosynthesis of Other Secondary Metabolites	1.59	0.01	1.54	0.00	0.05	0.884
Carbohydrate Metabolism	14.72	0.73	19.60	0.32	-4.88	0.039*
Cellular Processes and Signaling	10.30	0.26	0.84	0.00	9.46	<0.001**
Energy Metabolism	8.84	0.27	10.49	0.12	-1.65	0.222
Enzyme Families	20.35	4.63	10.33	0.67	10.02	0.304
Glycan Biosynthesis and Metabolism	3.06	0.05	4.41	0.04	-1.35	0.106
Lipid Metabolism	5.27	0.12	7.56	0.03	-2.29	0.036*
Metabolism of Cofactors and Vitamins	3.17	0.03	3.89	0.02	-0.72	0.165
Metabolism of Other Amino Acids	2.56	0.03	4.01	0.01	-1.45	0.012*
Metabolism of Terpenoids and Polyketides	3.67	0.02	3.98	0.01	-0.31	0.269
Nucleotide Metabolism	6.04	0.16	6.17	0.06	-0.12	0.914
Xenobiotics Biodegradation and Metabolism	4.29	0.06	7.06	0.10	-2.77	0.012*

Supplementary Table 3. Welch's t-test for predicted gene families associated with xenobiotics metabolism and biodegradation for captive and wild Andean bear samples. K-values were corrected for multiple hypothesis testing using Bonferroni correction. *P*-value < 0.05: *. Differences in mean relative abundance of each gene family and its standard deviation are shown.

**Xenobiotics biodegradation and metabolism pathways**	**Captive**		**Wild**			
	**Mean rel. freq. (%)**	**S.D.**	**Mean rel. freq. (%)**	**S.D.**	**Difference between means**	**p-values (corrected)**
1,1,1-Trichloro-2,2-bis(4-chlorophenyl)ethane_(DDT)_degradation	0.00	0.00	0.01	0.01	-0.01	0.63
Aminobenzoate_degradation	9.61	5.26	12.71	2.32	-3.09	4.14
Atrazine_degradation	0.40	1.07	1.73	0.68	-1.33	0.58
Benzoate_degradation	10.58	6.80	17.66	3.04	-7.08	0.76
Bisphenol_degradation	4.47	3.38	2.46	0.38	2.00	3.28
Caprolactam_degradation	4.81	4.05	8.42	0.65	-3.61	1.03
Chloroalkane_and_chloroalkene_degradation	9.69	5.12	7.35	0.90	2.35	5.46
Chlorocyclohexane_and_chlorobenzene_degradation	3.25	3.73	2.28	0.59	0.97	10.40
Dioxin_degradation	1.10	2.52	2.21	0.55	-1.12	5.84
Drug_metabolism_-_cytochrome_P450	5.13	3.40	6.46	0.68	-1.33	6.97
Drug_metabolism_-_other_enzymes	7.96	4.45	5.64	2.17	2.32	5.43
Ethylbenzene_degradation	1.20	1.63	2.31	0.45	-1.11	2.51
Fluorobenzoate_degradation	2.33	2.18	1.26	0.32	1.08	4.74
Metabolism_of_xenobiotics_by_cytochrome_P450	5.13	3.40	6.26	0.60	-1.13	8.38
Naphthalene_degradation	6.77	3.54	7.53	0.32	-0.76	11.80
Nitrotoluene_degradation	1.27	2.10	2.49	0.84	-1.22	4.10
Polycyclic_aromatic_hydrocarbon_degradation	18.75	30.83	3.51	0.85	15.25	4.65
Styrene_degradation	0.31	0.55	2.52	0.70	-2.21	0.02*
Toluene_degradation	6.62	5.51	5.81	2.07	0.81	14.68
Xylene_degradation	0.62	1.27	1.40	0.61	-0.78	3.98
